# Silencing microRNA-143 protects the integrity of the blood-brain barrier: implications for methamphetamine abuse

**DOI:** 10.1038/srep35642

**Published:** 2016-10-21

**Authors:** Ying Bai, Yuan Zhang, Jun Hua, Xiangyu Yang, Xiaotian Zhang, Ming Duan, Xinjian Zhu, Wenhui Huang, Jie Chao, Rongbin Zhou, Gang Hu, Honghong Yao

**Affiliations:** 1 Department of Pharmacology, School of Medicine, Southeast University, Nanjing, Jiangsu, China; 2 Jiangsu Key Laboratory of Neurodegeneration, Department of Pharmacology, Nanjing Medical University, 140 Hanzhong Road, Nanjing, Jiangsu, China; 3 Institute of Life Sciences, Key Laboratory of Developmental Genes and Human Disease, Southeast University, Nanjing, Jiangsu, China; 4 Virosis Laboratory, Key Laboratory of Zoonosis, Ministry of Education, Institute of Zoonosis, Jilin University, 5333 Xi An Road, Changchun, 130062, China; 5 Department of Molecular Physiology, Center for Integrative Physiology and Molecular Medicine, University of Saarland, Homburg D-66421, Germany; 6 Department of Physiology, School of Medicine, Southeast University, Nanjing, Jiangsu, China; 7 Institute of Immunology and the CAS Key Laboratory of Innate Immunity and Chronic Disease, School of Life Sciences and Medical Center, University of Science and Technology of China, Hefei, China

## Abstract

MicroRNA-143 (miR-143) plays a critical role in various cellular processes; however, the role of miR-143 in the maintenance of blood-brain barrier (BBB) integrity remains poorly defined. Silencing miR-143 in a genetic animal model or via an anti-miR-143 lentivirus prevented the BBB damage induced by methamphetamine. miR-143, which targets p53 unregulated modulator of apoptosis (PUMA), increased the permeability of human brain endothelial cells and concomitantly decreased the expression of tight junction proteins (TJPs). Silencing miR-143 increased the expression of TJPs and protected the BBB integrity against the effects of methamphetamine treatment. PUMA overexpression increased the TJP expression through a mechanism that involved the NF-κB and p53 transcription factor pathways. Mechanistically, methamphetamine mediated up-regulation of miR-143 via sigma-1 receptor with sequential activation of the mitogen-activated protein kinase (MAPK) and phosphatidylinositol-3′ kinase (PI3K)/Akt and STAT3 pathways. These results indicated that silencing miR-143 could provide a novel therapeutic strategy for BBB damage-related vascular dysfunction.

The blood-brain barrier (BBB) is a dynamic network that helps maintain CNS homeostasis by restricting the passage of toxic substances into the brain. Brain microvascular endothelial cells (BMECs) are the basic components of the BBB and play a critical role in maintaining the integrity of the BBB under physiological conditions^1^,^2^,^3^,^4^,^5^,^6^. BBB dysfunction has been demonstrated in various neurological disorders, including stroke, Alzheimer’s disease, and epilepsy, as well as in drug abuse^7^,^8^,^9^, which is a major social and health concern. Methamphetamine is a popular addictive pharmacological CNS psychostimulant, and its use is associated with multiple adverse neuropsychiatric reactions and with neurotoxicity in the dopaminergic and serotonergic systems of the brain^10^,^11^.

Methamphetamine exposure *in vivo* has been shown to disrupt the BBB^12^,^13^,^14^,^15^. Disruption of BBB integrity is not only a common consequence of the neuroinflammation induced by methamphetamine^16^, it also contributes to its progression. Protection of the cerebral endothelium is important for the therapy of methamphetamine-induced BBB damage. However, few interventions that target these processes have shown sufficient efficacy because the molecular mechanisms underlying methamphetamine-induced cerebral endothelial injury have not been well defined.

MicroRNAs (miRNAs) are short, evolutionarily conserved noncoding RNA molecules that are derived from much larger primary transcripts. Although the role of miRNAs in the regulation of cell proliferation, differentiation, migration and apoptosis has been previously recognized, an understanding of the importance of miRNAs in BBB integrity is just emerging. Recent studies revealed a critical role of miRNAs in controlling the function of the endothelial barrier of the brain under various conditions. For example, miR-29b has been reported to indirectly influence barrier function by targeting genes that regulate a wide range of events and may be a contributing factor in ischemic injury^17^. Additionally, miR-125a-5p plays a role in the BBB; this miRNA was shown to directly regulate barrier function in an *in vitro* BBB model and could reduce monocyte migration through a BBB cell layer *in vitr*o^18^. However, the association between miRNA and methamphetamine-induced brain endothelial dysfunction has not yet been systematically studied. In this study, we first demonstrated a significant increase in the level of miR-143 in the serum from methamphetamine abusers compared with healthy controls. miR-143 has a well-established role in the differentiation and proliferation of bovine intramuscular preadipocytes^19^. Moreover, miR-143 decreases cell growth by targeting syndecan-1 in melanoma^20^ and colon tumor cells^21^. Jordan *et al.* demonstrated that obesity-induced miR-143 overexpression (OE) inhibits insulin-stimulated Akt activation and impairs glucose metabolism^22^. However, the role of miR-143 in BBB integrity remains poorly understood. Consistent with the finding in serum from methamphetamine abusers, treatment of human brain microvascular endothelial cells (HBMECs) with methamphetamine increased the expression of miR-143. This finding encouraged us to examine its role in the integrity of the BBB in the context of methamphetamine abuse.

Computational algorithms, such as TargetScan, were employed to identify targets of miR-143, and p53 unregulated modulator of apoptosis (PUMA) was a predicted target. PUMA is one of the most common apoptosis inducers among the Bcl-2 homology domain 3 (BH3)-only subgroup of the Bcl-2 family^23^,^24^. PUMA was initially identified as a transcriptional target of p53 and as a mediator of DNA damage-induced apoptosis^25^,^26^. Although the function of PUMA in cell apoptosis has been extensively illustrated in various tissues, neither the involvement of PUMA in methamphetamine-induced BBB damage nor the regulation of PUMA expression by noncoding RNAs has been explored.

Here, we report that miR-143 is up-regulated in the brain microvessels of methamphetamine-treated mice and show that silencing miR-143 attenuates methamphetamine-induced BBB damage.

## Results

### Methamphetamine regulates miR-143 in the brain and in HBMECs

**Figure 1:**
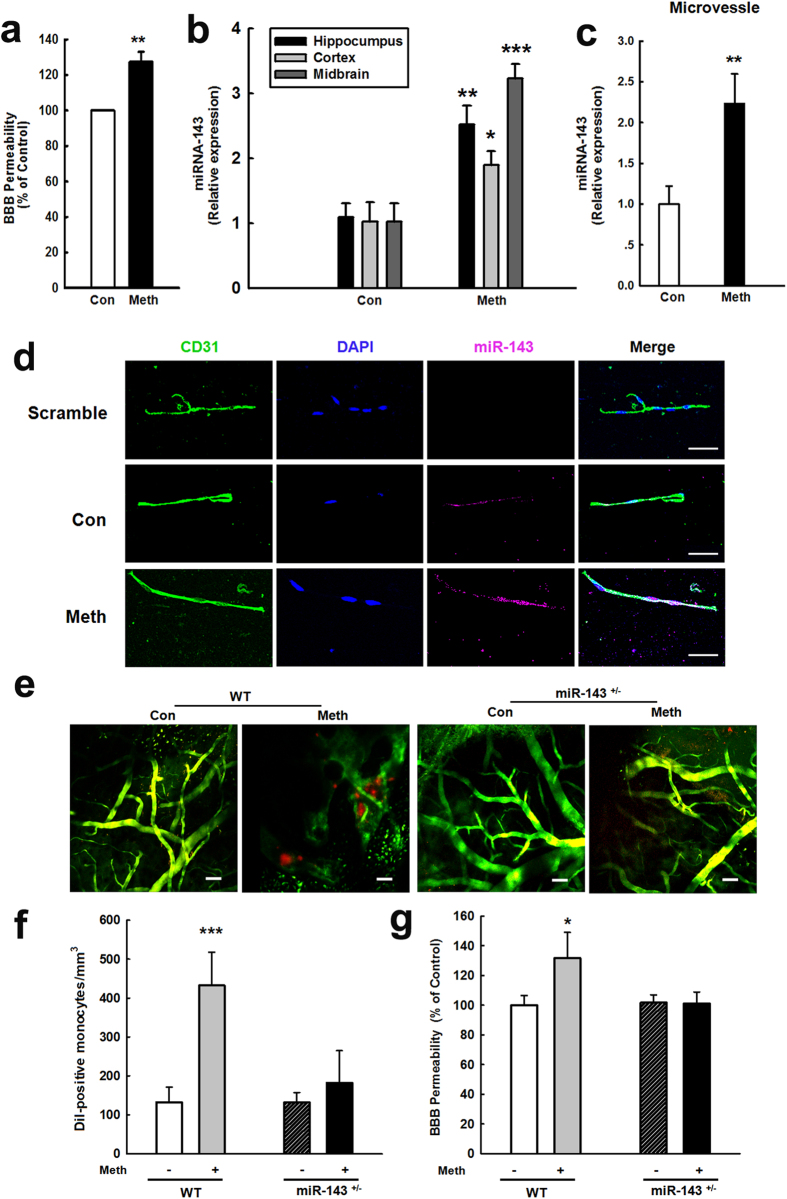
miR-143 is regulated by methamphetamine in the brain and in HBMECs. **(a**) BBB permeability was evaluated by measuring the amount of Evans blue extravasation via spectrophotometry of brain tissue at 620 nm. **(b,c)** Effect of methamphetamine on the mRNA expression of miR-143 in brain tissue from various brain regions, such as the hippocampus, cortex, striatum and midbrain and in microvessels in mice, as determined by real-time PCR. n = 6 animals/group. **p < 0.01 *vs.* control using Student’s t-test. **(d)** Fluorescence *in situ* hybridization of mature miR-143 in microvessels combined with immunostaining for the endothelial cell marker caveolin-1. Red, miR-143; green, caveolin-1; blue, DAPI. Scale bar = 20 μm. **(e,f)** Representative images from the TPLSM analysis of the methamphetamine-induced transmigration of monocytes from blood vessels in WT and miR-143^+/−^ mice. Scale bar = 50 μm. **(g)** BBB permeability was evaluated by measuring the amount of brain extravasation of Evans blue in WT and miR-143^+/−^ mice via spectrophotometry at 620 nm. n = 6 animals/group. *p < 0.05 and ***p < 0.001 *vs.* the WT control group using one-way ANOVA and the Holm-Sidak test. Meth, methamphetamine.

The finding that miR-143 was significantly increased in the serum from methamphetamine abusers compared with the control group (Supplementary Fig. S1) prompted us to investigate whether pathologic brain activity *in vivo* affects miR-143 levels. Methamphetamine administration caused BBB damage (Fig. 1a) and concomitant up-regulation of mature miR-143 in isolated microvessels (Fig. 1c) and in tissue from various brain regions, such as the hippocampus, cortex, striatum and midbrain (Fig. 1b). Moreover, fluorescence *in situ* hybridization (FISH) revealed that methamphetamine treatment increased the miR-143 expression in the isolated microvessels compared with that in the control group (Fig. 1d). To further confirm the role of miR-143 in methamphetamine-induced increase in BBB permeability, we examined monocyte migration from the blood in miR-143^+/−^ mice using *in vivo* two-photon laser scanning microscopy (TPLSM). Methamphetamine significantly increased the number of monocytes in WT mice but not in miR-143^+/−^ mice (Fig. 1e,f). The BBB leakage was further confirmed by the extravasation of Evans blue (Fig. 1g). The methamphetamine treatment increased the BBB permeability in WT mice but not in miR-143^+/−^ mice, demonstrating the role of miR-143 in regulating methamphetamine-mediated BBB damage *in vivo*.

### Silencing miR-143 ameliorated the increased permeability of the BBB and endothelial cells *in vivo*

**Figure 2:**
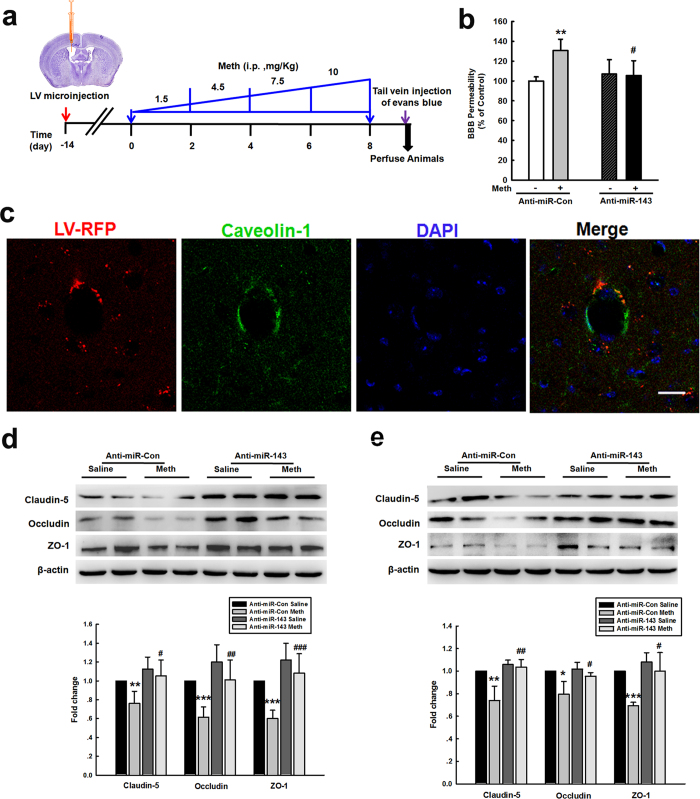
Silencing miR-143 ameliorated the increased permeability of the BBB and endothelial cells *in vivo.* **(a)** Schematic diagram depicting the procedure for the microinjection of the lentivirus into the brain ventricle. Two weeks after the lentivirus injection, the animals in the anti-control and anti-miR-143 lentivirus groups were intraperitoneally injected with either saline or methamphetamine (1.5 mg/kg, 4.5 mg/kg, 7.5 mg/kg, and 10 mg/kg) every day for a total of eight days according to the previously described dosing schedule. **(b)** The BBB permeability was evaluated by measuring the amount of brain extravasation of Evans blue in the animals microinjected with a lentivirus via spectrophotometry at 620 nm. **(c)** Representative images of microvessels in C57BL/6 mice microinjected with the RFP lentivirus. Representative images following microinjection of anti-miR-143 into the lateral ventricle. Caveolin-1 staining was conducted 2 weeks later. Green: caveolin-1; Red: RFP. Scale bar = 20 μm. **(d,e)** Microinjection of anti-miR-143 ameliorated the decreased TJP expression induced by methamphetamine in the cortex **(d)** and hippocampus **(e)**, as determined by western blot analysis. n = 6 animals/group. *p < 0.05, **p < 0.01 and ***p < 0.001 *vs.* the saline+anti-miR-control group; ^#^p < 0.05, ^##^p < 0.01 and ^###^p < 0.001 *vs.* the methamphetamine+anti-miR-control group using one-way ANOVA followed by the Holm-Sidak test. Meth, methamphetamine.

To validate the role of miR-143 *in vivo*, an anti-miR-143-RFP lentivirus was microinjected into the left lateral ventricle of mice, as illustrated in Fig. 2a. Methamphetamine treatment increased the BBB permeability in the anti-miR-control-injected group (Fig. 2b), but this effect was significantly ameliorated in the anti-miR-143-injected group. RFP was expressed in both the parenchyma and blood vessels, and a certain number of caveolin-positive cells co-localized with RFP (Fig. 2c). Our study indicated that methamphetamine administration significantly decreased the expression of tight junction proteins (TJPs) in the cortex and hippocampus in the anti-miR-control group, and this effect was significantly ameliorated in the anti-miR-143-microinjected mice (Fig. 2d,e)

### Silencing miR-143 ameliorated the increased permeability of the BBB and endothelial cells *in vitro*

**Figure 3:**
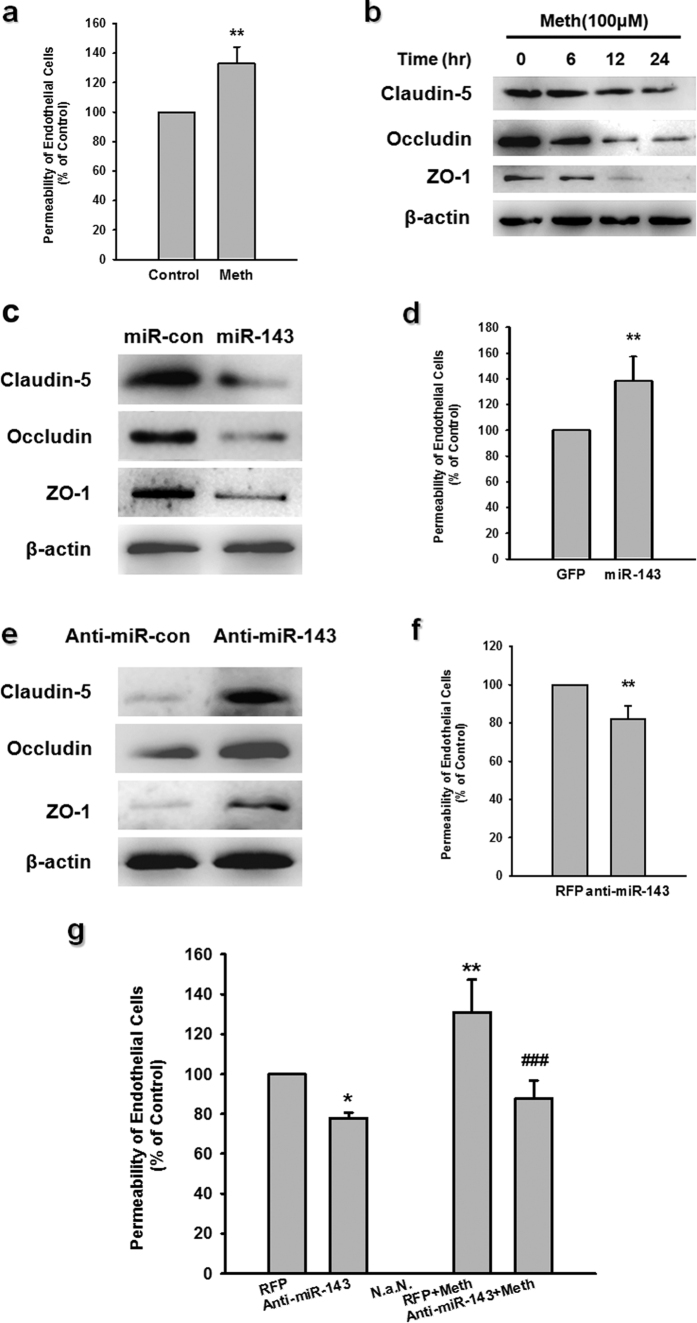
Effect of miR-143 on the permeability of endothelial cells *in vitro.* **(a)** Methamphetamine increased the permeability of the HBMECs. **(b)** Effect of methamphetamine on the expression of claudin-5, occludin, and ZO-1 in HBMECs evaluated using western blot analysis. **(c,d)** Effect of miR-143 on the expression of tight junction proteins **(c)** and the permeability of HBMECs **(d)**. **(e,f)** Effect of anti-miR-143 on the expression of tight junction proteins **(e)** and the permeability of HBMECs **(f)**. All data are presented as the mean ± SD of three individual experiments. **p < 0.01 *vs.* the control group using Student’s t-test. **(g)** Anti-miR-143 attenuated the methamphetamine-induced increase in the permeability of HBMECs. *p < 0.05 and **p < 0.01 *vs.* the anti-miR-con/control; ^###^p < 0.001 *vs.* the anti-miR-con/meth group using one-way ANOVA followed by the Holm-Sidak test. Meth, methamphetamine.

We next sought to explore the role of miR-143 in methamphetamine-mediated endothelial permeability *in vitro*. Exposing HBMECs to methamphetamine (100 μM) increased the endothelial permeability (Fig. 3a), an effect that was accompanied by a concomitant time-dependent decrease in the expression of TJPs (Fig. 3b). This concentration of methamphetamine failed to affect the viability of HBMECs (Supplementary Fig. S2), ruling out the possibility that the increased endothelial permeability was caused by cytotoxicity. These concentrations were chosen based on the concentration of methamphetamine (0.8–1.0 mM) in the postmortem brains of chronic abusers^27^,^28^.

The transduction of HBMECs with miR-143 decreased the expression of TJPs (Fig. 3c), an effect that was accompanied by a concomitant increase in endothelial permeability (Fig. 3d). Reciprocally, transducing HBMECs with anti-miRNA-143 had the opposite effect (Fig. 3e,f). We next examined the role of miR-143 in the methamphetamine-induced increase in endothelial cell permeability. The transduction of cells with the anti-miR-143 lentivirus reduced the methamphetamine-mediated increase in endothelial permeability (Fig. 3g). The extent of miR-143 overexpression and knockdown was assessed by real time-PCR (Supplementary Fig. S3).

### Role of PUMA in the effect of methamphetamine on BBB integrity

**Figure 4:**
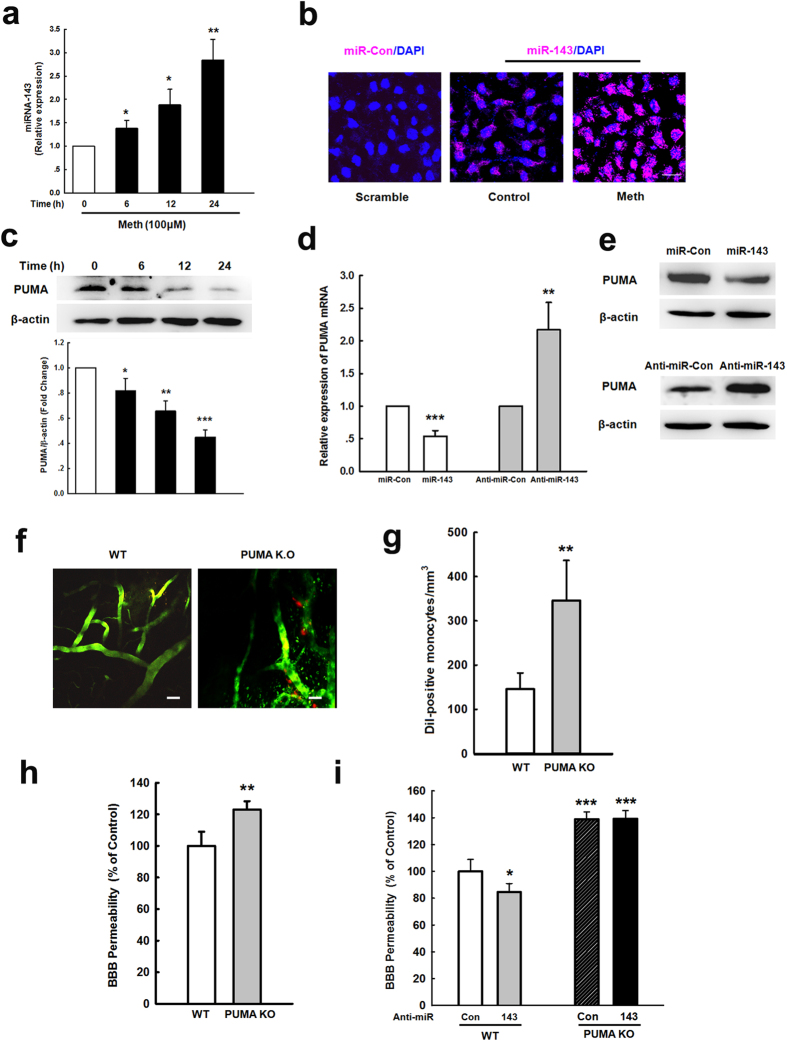
Role of PUMA in the effects of methamphetamine on BBB integrity. **(a)** Effect of methamphetamine on the mRNA expression of miR-143 in HBMECs, as determined by real-time PCR. **(b)** Fluorescence *in situ* hybridization of mature miR-143 in methamphetamine-treated HBMECs. Red, miR-143; blue, DAPI. Scale bar = 20 μm. (**c)** Methamphetamine decreased the PUMA expression in HBMECs, as determined by western blot analysis. **(d,e)** PUMA expression was evaluated at the mRNA **(d)** and protein **(e)** level in HBMECs that were transduced with the miR-control/miR-143 and the anti-miR-control/anti-miR-143 lentiviruses. All data are presented as the mean ± SD of three individual experiments. *p < 0.05, **p < 0.01 and ***p < 0.001 *vs.* the control group using one-way ANOVA followed by the Holm-Sidak test. **(f,g)** Representative images from the TPLSM analysis of the methamphetamine-induced migration of monocytes out of blood vessels in WT and PUMA KO mice. Scale bar = 50 μm. **(h)** The BBB permeability in WT and PUMA KO mice was determined by measuring the amount of brain extravasation of Evans blue by spectrophotometry at 620 nm. n = 6 animals/group. **p < 0.01 *vs.* the WT group using Student’s t-test. **(i)** The BBB permeability in WT and PUMA KO mice microinjected with the anti-miR-control/anti-miR-143 lentivirus was determined by measuring the amount of brain extravasation of Evans blue using spectrophotometry at 620 nm. n = 6 animals/group. **p < 0.01 and ***p < 0.001 *vs.* the WT mice microinjected with the anti-miR-control lentivirus using one-way ANOVA followed by the Holm-Sidak test. Meth, methamphetamine.

PUMA was predicted to have a conserved miR-143 binding site within its 3′-untranslated region (UTR) in most species. Methamphetamine treatment increased the expression of miR-143-3p (Fig. 4a) but not miR-143-5p (Supplementary Fig. S4a); this result was further confirmed by FISH (Fig. 4b) in HBMECs. Treating HBMECs with methamphetamine decreased the expression of PUMA (Fig. 4c), which was inversely correlated with the expression of miR-143. We constructed an miR-143-GFP lentivirus and an anti-miR-143-RFP lentivirus (Supplementary Fig. S4b). Consistently, in HBMECs, miR-143 decreased PUMA expression, whereas anti-miR-143 increased it, at both the mRNA (Fig. 4d) and protein (Fig. 4e) levels.

The role of PUMA, which is the target of miR-143, in BBB integrity has remained elusive; therefore, we examined the role of PUMA in BBB integrity. As shown in Fig. 4f,g, the number of Dil-labeled monocytes increased in PUMA KO mice compared with WT mice, as examined by TPLSM. This finding was further confirmed by the extravasation in an Evans blue assay (Fig. 4h). To further confirm the role of PUMA in the amelioration of BBB damage by anti-miR-143, PUMA KO mice were microinjected with anti-miR-143. As shown in Fig. 4i, microinjection of anti-miR-143 significantly decreased the BBB permeability in WT mice; however, anti-miR-143 failed to affect the BBB permeability in PUMA KO mice, suggesting a role of PUMA in the anti-miR-143-mediated protection of BBB integrity *in vivo*.

### miR-143 regulated the permeability of endothelial cells by targeting PUMA

**Figure 5:**
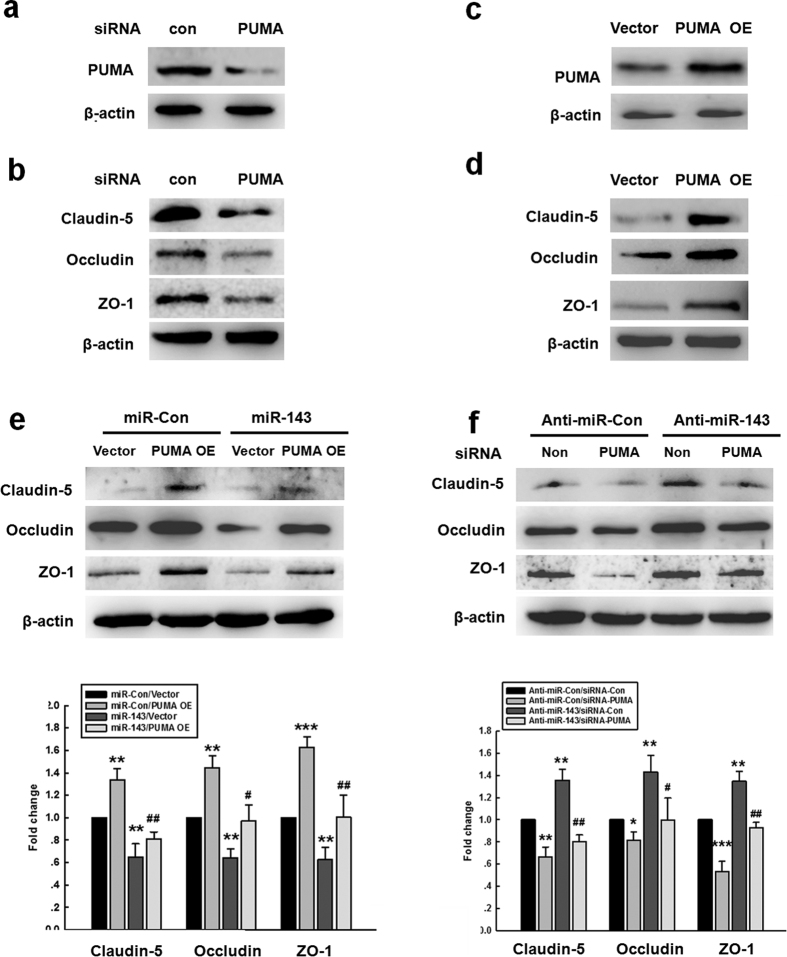
miR-143 regulated the permeability of endothelial cells by targeting PUMA. **(a,b)** The transduction of cells with the PUMA siRNA lentivirus decreased the PUMA expression and the expression of tight junction proteins (claudin-5, occludin, and ZO-1) in HBMECs. **(c,d)** The transduction of HBMECs with the PUMA OE lentivirus increased the expression of PUMA and tight junction proteins (claudin-5, occludin, and ZO-1) in HBMECs. **(e)** The transduction of cells with miR-143 failed to decrease the level of tight junction proteins in the cells co-transduced with the PUMA OE lentivirus, as determined by western blot analysis. **(f)** The transduction of cells with the PUMA siRNA lentivirus significantly inhibited the anti-miR-143-induced increase in the expression of tight junction proteins, as determined by western blot analysis. All data are presented as the mean ± SD of three independent experiments. *p < 0.05, **p < 0.01 and ***p < 0.001 *vs.* the miR-Con/Vector group or the anti-miR-Con/siRNA-Con group; ^+^p < 0.05 and ^##^p < 0.01 *vs.* the miR-143/Vector group or the anti-miR-143/siRNA-Con group using one-way ANOVA followed by the Holm-Sidak test. Meth, methamphetamine.

For further confirmation of whether miR-143 regulates the permeability of endothelial cells by targeting PUMA, HBMECs were transduced with PUMA siRNA or PUMA OE lentivirus and assessed for the expression of TJPs. Transduction of the cells with siPUMA significantly decreased the expression of PUMA (Fig. 5a), whereas transducing the cells with PUMA OE increased the level of PUMA (Fig. 5c). Intriguingly, transducing the cells with PUMA siRNA decreased the expression of TJPs (Fig. 5b), whereas transducing the HBMECs with PUMA OE increased the expression of TJPs (Fig. 5d). To determine whether the miR-143-mediated functional effects specifically depended on PUMA suppression, HBMECs were transduced with a PUMA OE construct that lacked the UTR (PUMA-Δ3′ UTR). Therefore, the PUMA expressed from the construct was not targeted by miR-143. In these HBMECs, miR-143 failed to decrease the expression of TJPs (Fig. 5e). Consistent with this finding, transducing the HBMECs with anti-miR-143 enhanced the expression of TJPs (Fig. 5f); this effect was ameliorated in cells that were co-transduced with PUMA siRNA.

### miR-143 induced the activation of the NF-κB and p53 transcription factors by targeting PUMA

**Figure 6:**
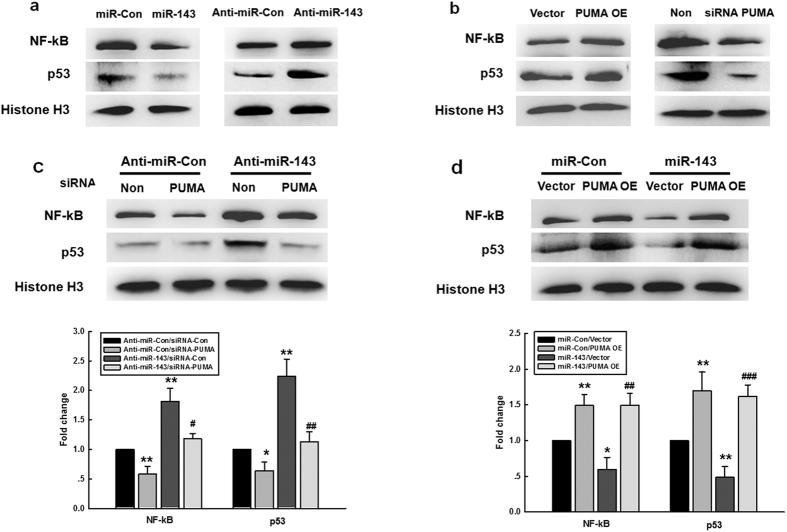
miR-143 induced the activation of the NF-κB and p53 transcription factors by targeting PUMA. **(a)** The nuclear translocation of p53 and NF-κB was decreased by the miR-143 lentivirus but increased by the anti-miR-143 lentivirus. **(b)** The translocation of p53 and NF-κB into the nucleus was decreased by PUMA siRNA decreased but increased by PUMA OE. **(c)** The PUMA siRNA lentivirus significantly inhibited the anti-miR-143-induced increase in the nuclear translocation of p53 and NF-κB. **(d)** The PUMA OE lentivirus significantly inhibited the miR-143-induced decrease in the nuclear translocation of p53 and NF-κB. All data are presented as the mean ± SD of three independent experiments. *p < 0.05 and **p < 0.01 *vs.* the anti-miR-Con/siRNA-Con group or the miR-Con/Vector group; ^#^p < 0.05, ^##^p < 0.01 and ^###^p < 0.001 *vs.* the anti-miR-143/siRNA-Con group or the miR-143/Vector group using one-way ANOVA followed by the Holm-Sidak test.

Next, we sought to identify the intracellular signaling pathways involved in the processes mediated by miR-143/PUMA. Transducing cells with miR-143 decreased the translocation of NF-κB and p53 into the nucleus, whereas transducing cells with anti-miR-143 increased the translocation of NF-κB and p53 into the nucleus (Fig. 6a). Intriguingly, the transduction of cells with PUMA siRNA decreased the translocation of NF-κB and p53 into the nucleus, whereas transduction with PUMA OE increased the nuclear translocation of these factors (Fig. 6b). The transduction of cells with the anti-miR-143 lentivirus increased the translocation of NF-κB and p53 into the nucleus, and this effect was ameliorated in cells that were co-transduced with the PUMA siRNA lentivirus (Fig. 6c). Reciprocally, the transduction of cells with the miR-143 precursor had the opposite effects; the transduction of cells with the miR-143 lentivirus decreased the translocation of NF-κB and p53 into the nucleus, and this effect was ameliorated in HBMECs that were co-transduced with the PUMA OE lentivirus (Fig. 6d).

### PUMA regulated the expression of TJPs via the NF-κB and p53 transcription factors

**Figure 7:**
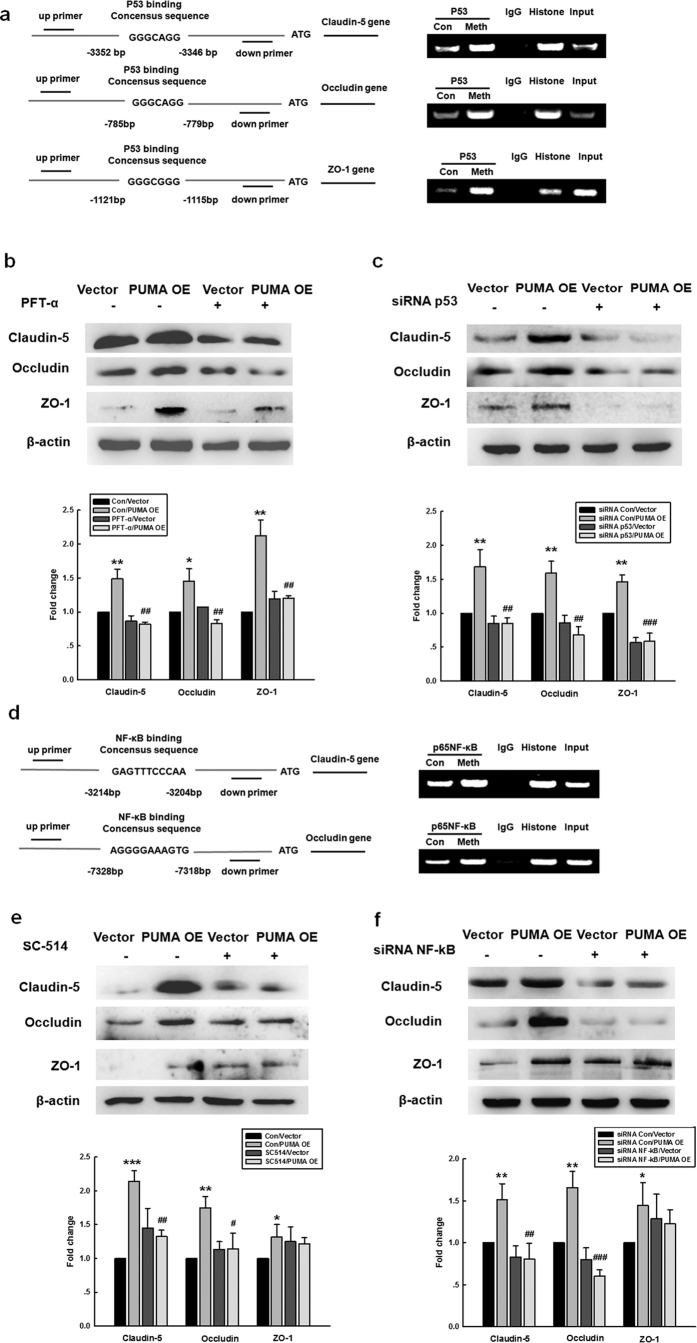
PUMA regulated the expression of tight junction proteins via pathways involving the transcription factors p53 and NF-κB. **(a)** ChIP assay demonstrating the methamphetamine-mediated binding of p53 to the promoters of tight junction proteins (claudin-5, occludin, and ZO-1). **(b,c)** Pretreatment of HBMECs with the p53 inhibitor PFT-α (10 μM) **(b)** or p53 siRNA **(c)** significantly decreased the PUMA OE-induced increase in the level of tight junction proteins. (**d)** ChIP assay demonstrating the methamphetamine-mediated binding of NF-κB to the promoters of claudin-5 and occludin, but not ZO-1. **(e,f)** Pretreatment of HBMECs with the NF-κB inhibitor SC-514 (10 μM) **(e)** or NF-κB siRNA **(f)** significantly decreased the PUMA OE-induced increase in the expression of claudin-5 and occludin but did not affect the expression of ZO-1. All data are presented as the mean ± SD of three independent experiments. *p < 0.05, **p < 0.01, and ***p < 0.001 *vs.* the Con/vector group; ^#^p < 0.05, ^##^p < 0.01 and ^###^p < 0.001 *vs.* the group treated with vector and the inhibitor or siRNA using one-way ANOVA followed by the Holm-Sidak test. Meth, methamphetamine.

We next investigated the role of downstream transcription factors (NF-κB *vs.* p53) in the miR-143-mediated expression of TJPs. As predicted using Primer 3.0 (Fig. 7a, left panel), putative p53 binding sites are located within the promoter regions of claudin-5, occludin, and ZO-1. Methamphetamine treatment of HBMECs increased the binding of p53 to the promoter regions of claudin-5, occludin and ZO-1 (Fig. 7a, right panel). Pretreatment of HBMECs with the p53 inhibitor PFT-α (5 μM) significantly decreased the PUMA OE-mediated increase in the expression of TJPs (Fig. 7b), and this result was further confirmed using p53 siRNA (Fig. 7c). In addition to the putative p53 binding sites, putative NF-κB binding sites were identified upstream of the claudin-5 and occludin promoter sequences; however, a putative NF-κB binding site was not found upstream of the ZO-1 promoter sequence (Fig. 7d, left panel). Methamphetamine treatment increased the binding of NF-κB to the promoter region of claudin-5 and occludin (Fig. 7d, right panel). Pretreatment of HBMECs with the IKK-2 inhibitor SC-514 (5 μM) significantly decreased the PUMA OE-mediated enhancement of the expression of claudin-5 and occludin but did not affect the expression of ZO-1 (Fig. 7e); this result was further confirmed through the use of NF-κB siRNA (Fig. 7f).

### The sigma-1 receptor/MAPK/STAT3 pathway was involved in the methamphetamine-induced expression of miR-143

**Figure 8:**
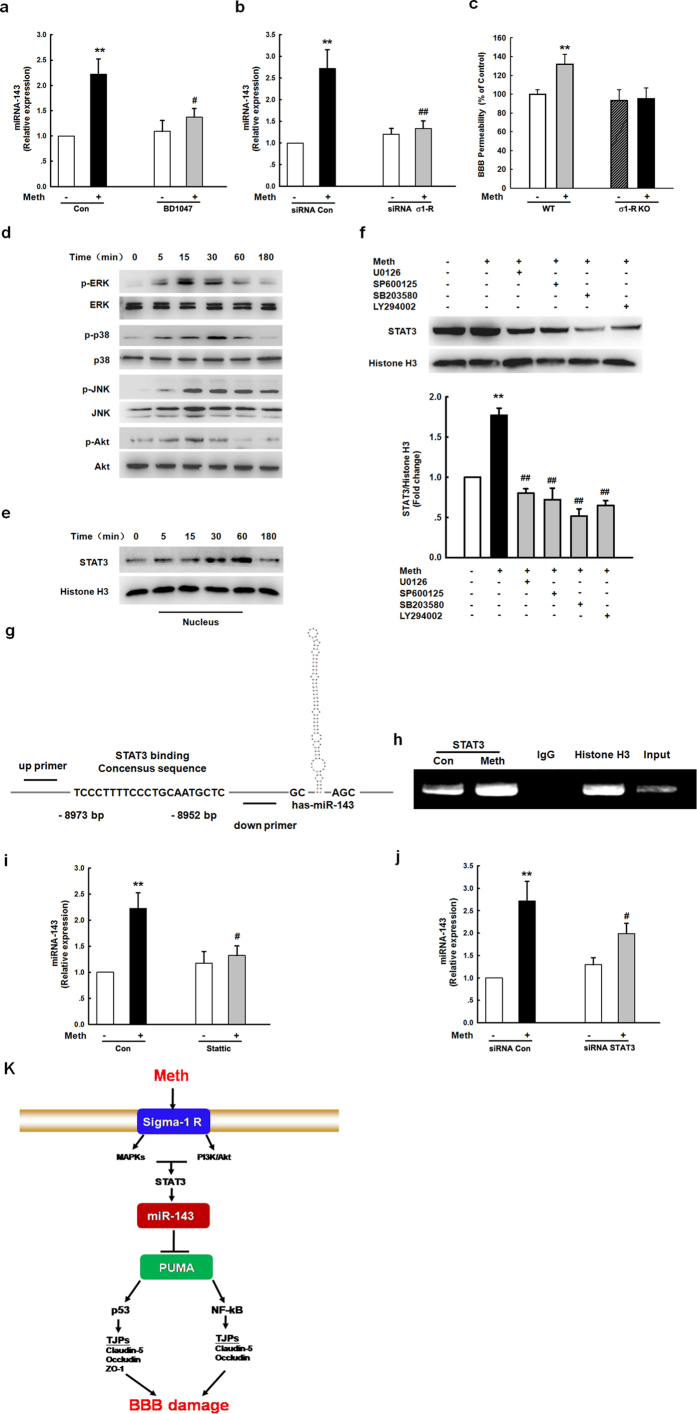
The σ-1R/MAPK/STAT3 pathway was involved in the methamphetamine-induced expression of miR-143. **(a,b)** Pretreatment of HBMECs with a σ-1R inhibitor (BD1047, 10 μM) **(a)** or σ-1R siRNA **(b)** significantly inhibited the methamphetamine-induced increase in the expression of miR-143, as determined by real-time PCR. **(c)** Administering methamphetamine to the animals damaged the BBB in WT mice but not in σ-1R KO mice. **(d,e)** Effect of methamphetamine on the activation of the MAPK and PI3K/Akt cell signaling pathways. **(d)** Increase in the translocation of STAT3 into the nucleus **(e)**. **(f)** Pretreatment of HBMECs with an MEK inhibitor (U0126, 10 μM), JNK inhibitor (SP600125, 10 μM), p38 inhibitor (SB203580, 10 μM), and PI3K inhibitor (LY294002, 5 μM) inhibited the methamphetamine-mediated translocation of STAT3 into the nucleus. **(g,h)** ChIP assay demonstrating methamphetamine-mediated binding of STAT3 to the miR-143 promoter. **(I,j)** Pretreatment of HBMECs with a STAT3 inhibitor (Stattic, 1 μM) **(i)** or STAT3 siRNA **(j)** significantly inhibited the methamphetamine-induced increase in the expression of miR-143. **(k)** miR-143 regulation of BBB integrity via the targeting of PUMA, the subsequent distinct downstream activation of the p53 and NF-κB pathways, and the cooperative expression of TJPs in endothelial cells. Mechanistically, methamphetamine mediated the up-regulation of miR-143 via the sigma-1 receptor with sequential activation of mitogen-activated protein kinases (MAPKs) and the phosphatidylinositol-3′ kinase (PI3K)/Akt and STAT3 pathways. All data are presented as the mean ± SD of three independent experiments. **p < 0.01 *vs.* the control, ^#^p < 0.05 and ^##^p < 0.01 *vs.* the methamphetamine-treated group using one-way ANOVA followed by the Holm-Sidak test. Meth, methamphetamine.

We next wanted to investigate the mechanism(s) underlying the expression of miR-143. The sigma-1 receptor (σ**-**1R), which belongs to the non-opioid receptor family, binds to a diverse array of psychotropic drugs, including methamphetamine^11^,^29^,^30^. The methamphetamine-mediated induction of miR-143 was significantly attenuated in cells that were pretreated with the known σ**-**1R antagonist BD1047 (Fig. 8a) and those that were transfected with σ**-**1R siRNA (Fig. 8b). Moreover, methamphetamine administration caused BBB damage in WT mice but failed to cause BBB damage in σ**-**1R^−/−^ mice (Fig. 8c).

Methamphetamine treatment transiently increased the phosphorylation of ERK, JNK and p38 MAPK (Fig. 8d). Putative STAT3 binding sites are predicted upstream of the miR-143 promoter sequence, and methamphetamine treatment increased the nuclear translocation of STAT3 (Fig. 8e). Pretreatment with a MEK1/2 inhibitor (U0126), a p38 inhibitor (SB203580), a JNK inhibitor (SP600125), or a PI3K inhibitor (LY294002) inhibited the methamphetamine-induced translocation of STAT3 into the nucleus (Fig. 8f). Under natural chromatin conditions, STAT3 binds to the miR-143 promoter (Fig. 8g). Methamphetamine treatment increased the binding of STAT3 to the miR-143 promoter in HBMECs (Fig. 8h), and the methamphetamine-mediated induction of miR-143 was significantly attenuated by pretreating the cells with the known STAT3 inhibitor Stattic (Fig. 8i); this result was further confirmed using STAT3 siRNA (Fig. 8j).

## Discussion

Methamphetamine is known to have a deleterious effect on BBB integrity; however, the complex network underlying the regulation of the effects of methamphetamine by miRNAs and their targets has not yet been fully elucidated. Silencing miR-143 in a genetic animal model or via an anti-miR-143 lentivirus ameliorated the BBB damage induced by methamphetamine. To our knowledge, these are the first *in vivo* data to show that inhibition of a single mature miRNA can alter the integrity of the BBB. Mechanistically, our study provides new insight into the role of miR-143 in the regulation of BBB integrity by demonstrating its targeting of PUMA and the subsequent distinct downstream activation of the p53 and NF-κB pathways and the altered expression of TJPs in endothelial cells (Fig. 8k). The regulation of miR-143/PUMA expression may be a therapeutic intervention to help regulate BBB integrity in the context of drug abuse.

Although previous studies indicated that miR-143 exerts its tumor-suppressive function by targeting oncogenes, such as syndecan-1, KRAS and C/EBPα^19^,^20^,^31^, the role of miR-143 in the maintenance of BBB integrity has not been determined. In this study, we demonstrate that methamphetamine treatment increased the expression of miR-143 both *in vitro* and *in vivo*, providing a biological basis for the interaction between methamphetamine and miR-143. The *in vivo* importance of these findings was further corroborated in mice: we demonstrated that methamphetamine increased the permeability of the BBB in WT mice but not miR-143^+/−^ mice. Consistent with previous findings regarding the role of miR-143 in endothelial cells^32^, miR-143 was involved in the response of endothelial cells to shear stress. Moreover, Climent *et al.* demonstrated that miR-143 provides a means of communication between smooth muscle cells and endothelial cells to regulate the vessel stabilization properties of endothelial cells^33^. In addition to being passed from smooth muscle cells to endothelial cells, miR-143 was also shuttled in extracellular vesicles derived from endothelial cells, and miR-143 regulated the expression of differentiation-related target genes in the smooth muscle cells^34^. Although miR-143 reportedly plays a fundamental role in smooth muscle differentiation during physiological and pathological events^35^,^36^,^37^,^38^,^39^, to our knowledge, this is the first *in vitro* and *in vivo* demonstration of the involvement of miR-143 in the expression of TJPs.

Another novel finding of this study was that PUMA, a newly identified target of miR-143, mediated the expression of TJPs in endothelial cells. As an apoptosis inducer, PUMA has been shown to be involved in the apoptosis of endothelial cells^40^,^41^. In the current study, methamphetamine failed to affect the apoptosis of endothelial cells (Supplementary Fig. S5), ruling out the possibility that the involvement of PUMA could be attributed to its role as an inducer of apoptosis. In our study, knockdown of PUMA decreased the expression of TJPs, whereas PUMA OE increased TJP expression. This finding, along with a previous report showing a significant decrease in the number of endothelial cells in the retina in PUMA KO mice compared with that in WT mice^42^, suggests a novel function of PUMA in BBB integrity. However, our findings are not consistent with those of a previous study indicating that PUMA knockdown inhibited cell apoptosis and restored BBB integrity via the endoplasmic reticulum following subarachnoid hemorrhage^43^. Distinct roles for PUMA in different cellular environments could provide an explanation for this inconsistency. As shown here, the transduction of HBMECs with either PUMA siRNA or the PUMA OE lentivirus failed to induce apoptosis of the HBMECs regardless of whether methamphetamine treatment was administered. Therefore, understanding the distinct roles of PUMA could help advance our understanding of the detailed mechanisms underlying the diverse functions of PUMA in different contexts.

Our study provides new insight into the role of miR-143 in the regulation of BBB integrity by demonstrating its targeting of PUMA and the subsequent distinct downstream activation of the p53 and NF-κB pathways and the altered expression of TJPs in HBMECs. PUMA was initially identified as a transcriptional target of p53 and a mediator of DNA damage-induced apoptosis^25^,^26^. Interestingly, the results of our study indicated that PUMA OE increased the translocation of p53 into the nucleus; future studies are needed to further investigate the mechanisms by which PUMA OE induced the translocation of p53 into the nucleus. In other systems, miR-18a has been shown to increase the permeability of the blood–tumor barrier via RUNX1-mediated down-regulation of the tight junction-related proteins ZO-1, occludin and claudin-5^44^. Different pathways are involved in the expression of TJPs; for example, the tight junction CLDN2 gene is a direct target of the vitamin D receptor^45^. The activation of matrix metalloproteinases also contributed to the decreased expression of TJPs^46^. Although a previous study indicated that p53 increased endothelial permeability via the induction of endothelial cell apoptosis^47^, our study provided direct evidence of the role of p53 in the regulation of TJP expression. In addition to the effects on p53, the activation of NF-κB was also involved in the PUMA OE-mediated expression of claudin-5 and occludin, but NF-κB did not influence the expression of ZO-1. Our findings are not consistent with those of a previous study that showed that the occludin and ZO-1 expression levels were decreased in the dextran sodium sulfate-induced model of colitis; this effect was significantly inhibited by pyrrolidine dithiocarbamate (PDTC), an inhibitor of NF-κB^48^. This inconsistency could be explained if NF-κB activation was able to decrease the expression of TJPs via other indirect molecules, despite NF-κB not being predicted to bind to the ZO-1 promoter. As demonstrated in a previous study, matrix metalloproteinase activation lies downstream of NF-κB activation, which decreases TJP expression^46^.

The use of pharmacological and genetic approaches to further dissect the signaling pathways involved in the methamphetamine-mediated induction of miR-143 revealed the activation of σ-1R and the downstream STAT3 pathway. The transcription factor STAT3 has emerged as a major regulatory transcription factor for miR-143, as predicted. In agreement with our findings, the requirement for activation of both MAPK/Akt and STAT3 has also been reported for the production of CCL2 in human umbilical vein endothelial cells^49^. A previous study also demonstrated that miR-143 and STAT3 regulated the expression of hexokinase 2 in breast cancer cells^50^. In addition to STAT3, Kruppel-like factor 2 is involved in the expression of miR-143 in endothelial cells^34^. To our knowledge, this is the first study to provide direct evidence of the regulatory role of STAT3 in the expression of miR-143.

Taken together, these results indicate that miR-143/PUMA mediates a regulatory pathway that is critical for the maintenance of BBB integrity. Specific regulation of miR-143/PUMA could be a potential therapeutic target for the treatment of BBB damage.

## Materials and Methods

### Reagents

The miR-143 lentivirus, anti-miR-143 lentivirus and PUMA siRNA lentivirus were purchased from HANBIO (Shanghai, China). The PUMA OE plasmid (pHA-PUMA, Plasmid #16588) was obtained from Addgene (USA). Based on the sequence of this PUMA OE plasmid, an expression construct encoding the entire PUMA coding sequence but lacking the 3′ UTR, thus yielding an mRNA resistant to miRNA-mediated suppression, was prepared by HANBIO (Shanghai, China). Methamphetamine was ordered from the National Institute for the Control of Pharmaceutical and Biological Products (Beijing, China). Control siRNA, human STAT3 siRNA, σ-1R siRNA, NF-κB siRNA and pifithrin-α hydrobromide (PFT-α) were obtained from Santa Cruz Biotechnology (Dallas, TX, USA). The specific MEK1/2 inhibitor U0126, the JNK inhibitor SP600125, the p38 inhibitor SB203580, and the PI3K inhibitor LY294002 were purchased from Calbiochem (San Diego, CA). The IKK-2 inhibitor SC-514 was purchased from Sigma Chemicals (St. Louis, MO).

### Cell culture and treatments

The HBMECs were purchased from ScienCell (Carlsbad, CA, USA), cultured in endothelial medium (provided by ScienCell) and were used at passages 4–14. The cell culture dishes were coated with poly-L-lysine (2 μg/cm^2^). Cultured cells are negative for HIV-1, HBV, HCV, mycoplasma, bacteria, yeast, and fungi. siRNA for NF-κB, p53, σ**-**1R and STAT3 resulted in efficient knockdown of the corresponding protein (Supplementary Fig. S6). As shown in a previous study^51^, the concentrations of U0126, SP600125, SB203580 and LY294002 used in the present study did not affect the viability of HBMECs. PFT-α (5 μM) and SC-514 (5 μM) also did not affect the cell viability, as shown in Supplementary Fig. S7.

### Assessment of BBB integrity *in vivo*

As described in our previous studies^51^,^52^, BBB integrity was evaluated in C57BL/6 or PUMA KO mice following the lentivirus injection. The use of C57BL/6 or PUMA KO mice was approved and complied with the guidelines of Institutional Animal Care and Use Committee (IACUC) of Southeast University (approval ID: SYXK-2010.4987). For microinjection, 24 eight-week-old C57BL/6N mice were divided into the following four groups (n = 6 in each group; male): (1) saline+anti-miR-control; (2) methamphetamine+anti-miR-control; (3) saline+anti-miR-143; and (4) methamphetamine+anti-miR-143. The lentivirus (2 μl of 10^9^ viral genomes μl^−1^, HANBIO, Shanghai, China) was microinjected into the right lateral ventricle using the following microinjection coordinates: 0.34 mm caudal of bregma and 1.0 mm lateral of the sagittal midline, at a depth of 2.2 mm below the skull surface.

Two weeks later, the animals received eight injections of 1.5 to 10 mg/kg methamphetamine or saline. This dosing schedule was chosen based on the fact that a common pattern in most methamphetamine abusers is an initial use of lower doses with progressive increases to higher doses and eventually multiple daily administrations. Therefore, to simulate this pattern of methamphetamine exposure, we adapted a safe escalating dosing regimen that does not produce potentially lethal hyperthermia in methamphetamine-treated animals according to a previous studies^53^,^54^. The mice were injected intraperitoneally with incrementally increasing doses on alternating days (i.p, 1.5 mg/kg on days 1–2, 1 time/day; 4.5 mg/kg on days 3–4, 1 time/day; 7.5 mg/kg on days 5–6, 1 time/day; and 10 mg/kg on days 7–8, 4 times/day). On days 7–8, once the animals reached a total dose of 10 mg/kg, the methamphetamine was injected every 2 h for a total of four times/day. The control group was injected with vehicle in the same volume that was used for the methamphetamine treatments. On day 9, the animals were injected in the tail vein with 200 μl of Evans blue (2%, 4 ml/kg; Sigma) in PBS, which was allowed to circulate for 40 min. The mice were then anesthetized with isoflurane in oxygen and perfused with 30 ml of heparinized saline. The brains were then harvested and homogenized in PBS (1:10 g/v). The homogenates were precipitated in 15% trichloroacetic acid (1:1 v/v) and centrifuged at 1,000 g for 10 min. The pH was adjusted by adding 125 μl of 5 M sodium hydroxide to 500 μl supernatant aliquots. Evans blue was measured spectrophotometrically at 620 nm.

### Bone marrow-derived monocyte (BMM) isolation and Dil staining

Cultured BMMs were 98% CD11b^+^, as demonstrated by flow cytometry in our previous study^55^. Cultured cells were negative for HIV-1, HBV, HCV, mycoplasma, bacteria, yeast, and fungi. To evaluate monocyte migration, monocytes were washed with PBS and fluorescently labeled with 10 μM Dil (Beyotime, China) for 10 min at room temperature according to the manufacturer’s instructions (Sigma).

### *In vivo* two-photon laser scanning microscopy (TPLSM) for monocyte migration

For studies of monocyte migration, animals were injected with Dil-labeled BMM at a concentration at 10^7^ cells/100 μl through the tail vein. Twenty-four hours following cell infusion, the animals were subjected to TPLSM for the detection of monocyte migration according previous studies^56^,^57^. FITC-dextran (70,000 molecular weight, Sigma) was intravenously injected to visualize the cortical vasculature. In brief, mice were deeply anesthetized with an intraperitoneal injection of ketamine and xylazine. A sterile surgical blade was used to make a midline incision of the scalp to expose the skull. The head was glued with a head plate and then fixed to the holding plate using two lateral blocks. Afterward, screws were tightened to immobilize the head plate. Under a dissection microscope, a high-speed micro-drill was used to thin a circular area of the skull over the area of interest to a thickness of approximately 30 μm.

Image stacks of Dil-labeled monocytes were obtained using a two-photon laser scanning microscope (excitation source: Chameleon Ultra I Hands-Free Ti:Sapphire Ultrafast Laser). A long working distance (2 mm) water-immersion objective (20x, NA = 1.0) was used to measure the monocyte migration into the parenchyma through mouse brain vessels. The images were taken at an 8-bit depth with a resolution of 1024×1024 pixels. Monocyte migration was measured at each time point by transillumination intravital microscopy. Cortical brain vessels 20–40 μm in diameter and 100–150 μm in depth below the cortical surface were selected for two-photon imaging. Z-stack images of 50-μm thick tissue sections were collected at 2 μm steps, and three-dimensional pictures were constructed by ZEN2011 Imaging Software (ZEISS) to calculate the number of monocytes that migrated into the parenchyma.

### Isolation of brain microvessels

For the treatments with methamphetamine or saline, mice were perfused under anesthesia, and the brains were removed and immediately immersed in ice-cold isolation buffer according to the procedures of our previous study^58^.

### Western blot analysis

Proteins were extracted in RIPA lysis buffer (Beyotime, Shanghai, China), separated on sodium dodecyl sulfate polyacrylamide gels (8% and 12%) and electrophoretically transferred onto polyvinylidene fluoride membranes. The membranes were blocked with 5% non-fat dry milk in Tris-buffered saline with Tween-20, probed with antibodies recognizing p-ERK/ERK (1:1,000, Cell Signaling, #9101S/#9107S), p-JNK(1:1,000, Santa Cruz, sc-6254)/JNK(1:1,000, Cell Signaling, #9252S), p-p38/p38(1:1,000, Cell Signaling, #9211S/#9212S), p-AKT/AKT (1:1,000, Cell Signaling, #9271S/#9272S), NF-κB p65 (1:1,000, Cell Signaling, #3033S), STAT3 (1:1,000, Cell Signaling, #12640S), histone H3 (1:1,000, Cell Signaling, #9715S), claudin-5 (1:1,000, Abcam, ab15106), occludin (1:1,000, Life Technology, #33–1500), ZO-1 (1:1,000, Life Technology, #402300), p53 (1:1,000, Santa Cruz, sc-6243), PUMA (1:1,000, Santa Cruz, sc-28226) and β-actin (1:1,000, Bioworld, BS6007M) overnight at 4 °C, and then incubated with a horseradish peroxidase-conjugated goat anti-mouse/rabbit IgG secondary antibody (1:2,000, Cell Signaling, #7076P2/#7074P2). A Microchemi 4.2^®^ (DNR, Israel) digital image scanner was used for detection, and the band intensity was quantified using ImageJ software (NIH).

### Real-time PCR

Total RNA isolated from cells was subjected to reverse transcription using the PrimeScript RT Master Mix Kit (TaKaRa, Japan). Real-time PCR analysis was performed for PUMA (5′-GTCAAGAAAACGCACTCGTGG-3′ forward and 5′-GACCCCTATTTTTATGGGGCCAA-3′ reverse) and GAPDH (5′-CAATGACCCCTTCATTGACC-3′ forward and 5′-TTGATTTTGGAGGG ATCTCG-3′ reverse) as previously described^51^,^58^,^59^.

### Statistical analysis

Data are expressed as the mean ± SD. The significance of differences between controls and samples treated with various drugs was determined by one-way ANOVA followed by post hoc least significant difference (LSD) tests. Values of p < 0.05 were considered statistically significant.

## Additional Information

**How to cite this article**: Bai, Y. *et al.* Silencing microRNA-143 protects the integrity of the blood-brain barrier: implications for methamphetamine abuse. *Sci. Rep.* **6**, 35642; doi: 10.1038/srep35642 (2016).

## Supplementary Material

Supplementary Information
