# Supplementary material for: Silencing microRNA-143 protects the integrity of the blood-brain barrier: implications for methamphetamine abuse
Source: Sci Rep. 2016 Oct 21;6:35642. doi: 10.1038/srep35642 (PMC5073292; doi:10.1038/srep35642)
Supplement: Supplementary Information [file srep35642-s1.pdf]

Silencing microRNA-143 protects the integrity of the blood-brain barrier: implications for methamphetamine abuse

Ying Bai[1], Yuan Zhang[1], Jun Hua[2], Xiangyu Yang[3], Xiaotian Zhang[1], Ming Duan[4], Xinjian Zhu[1], Wenhui Huang[5], Jie Chao[6], Rongbin Zhou[7], Gang Hu[2], Honghong Yao[1,3*]

Supplementary Information

Animals

The miR-143 mutant mice (Mutant Mouse Resource & Research Centers supported by the NIH; https://www.mmrrc.org/catalog/cellLineSDS.php?mmrrc_id=34438) were generated using a standard miRNA KO targeting vector containing the PGK-EM7 promoter puroDtk selection cassette flanked by recombinase sites as described in our recent published study[1].

The PUMA KO mice (male, 6-8 weeks of age) were kindly shared by Dr. Gerard Zambetti of St. Jude's Children's Research Hospital, Inc. and by Dr. Tao Cheng from State Key Laboratory of Experimental Hematology, Institute of Hematology and Blood Disease Hospital, Chinese Academy of Medical Sciences and Peking Union Medical College. The σ-1R KO mice (male, 6-8 weeks of age) obtained from the Jackson Laboratory were backcrossed over

10 generations to a C57BL/6N inbred background. The C57BL/6N mice (male, 6-8 weeks of age) were purchased from the Comparative Medicine Centre, Yangzhou University (Yangzhou, China). All animals were housed under conditions of constant temperature and humidity on a 12-h light, 12-h dark cycle, with lights on at 07:00 h. Food and water were available *ad libitum*. Animals were randomly assigned to different treatments. Before the animals were perfused, they were deeply anesthetized via an overdose of isoflurane followed by pneumothorax. All animal procedures were approved by Institutional Animal Care and Use Committee (IACUC) of Southeast University (approval ID: SYXK-2010.4987).

MicroRNA array in serum from human subjects

The human serum samples were obtained from abstinent methamphetamine-dependent patients (n=17) at the Zhenjiang Jurong Detoxification Institute and age-matched healthy individuals (n=13) at Zhongda hospital affiliated with Southeast University. The serum was collected in sterile glass tubes at 8:00 am after overnight fasting. Total RNA containing small RNA was extracted from the serum using the Trizol reagent (Invitrogen) and purified with an mirVana miRNA Isolation Kit (Ambion, Austin, TX, USA). Total RNA was isolated using the miRNeasy kit, and miRNAs were reverse transcribed using the Megaplex Primer Pools (Human Pools A and B) from Applied Biosystems. The expression profile of miRNAs in each sample was

determined using the Human Taqman miRNA Arrays A and B (Applied Biosystems). The collection and subsequent biochemical analyses of human serum were approved and supervised by IEC for Clinical Research of Zhongda Hospital, Affiliated with Southeast University.

Culture of primary mouse astrocytes

Primary mouse astrocytes were obtained from postnatal (P1 to P2) C57BL/6N mice, which were purchased from the Comparative Medicine Centre, Yangzhou University. After the membranes and large blood vessels were dissociated, the dissected brain cortices in PBS supplemented with brain tissues were digested by trypsin-EDTA (Gibco, 25200056). Whole brains of mice were dissected and mechanically dissociated using gauze to remove the membranes and large blood vessels. Brain tissues were digested by Trypsin-EDTA; then, the cells were plated on poly-L-lysine pre-coated cell culture flasks in Dulbecco's modified Eagle's medium (DMEM) supplemented with FBS (10% v/v) and penicillin/streptomycin (1% v/v). Cultures were maintained in a humidified chamber (37°C, 5% CO_2 incubator). After 7 to 10 days, the astrocytes were harvested by trypsinization.

Fluorescence *in situ* hybridization (FISH) combined with immunostaining

The cells or microvessels were permeabilized with 0.3% Triton X-100 in PBS for 15 min, pre-hybridized in hybridization buffer (50% formamide, 10 mM

Tris-HCl [pH 8.0], 200 μg ml^{-1} yeast tRNA, 1X Denhardt's solution, 600 mM NaCl, 0.25% SDS, 1 mM EDTA, and 10% dextran sulfate) for 1 h at 37°C. Then, the coverslips or slides were heated to 65°C for 5 min in hybridization buffer containing 2 nM of a commercially available digoxigenin-labeled miR-143 probe (Exiqon), and hybridization was allowed to occur at 37°C overnight. The next day, the coverslips or slides were washed three times in 2X SSC and twice in 0.2X SSC at 42°C and then blocked with 1% BSA and 3% normal goat serum in PBS for 1 h at room temperature. The samples were then incubated with a horseradish peroxidase-conjugated anti-digoxigenin antibody (1:200, Roche Diagnostics GmbH, Mannheim, Germany, #11207733910) overnight at 4°C. After the coverslips or slides were washed three times with TBS, they were incubated with a TSA Cy5 kit (PerkinElmer, Waltham, MA, USA) for 10 min at room temperature. For microvessels, the slides were washed with 0.1% Tween-20 in PBS, washed twice in PBS and washed once in diethylpyrocarbonate (DEPC) water, blocked with 1% BSA and 3% normal goat serum in 1X PBS for 1 h at room temperature and incubated with CD31 antibody (1:250, Santa Cruz, sc-1506) overnight at 4°C. The slides were washed twice with PBS and incubated with Alexa Fluor 488-conjugated goat anti-rabbit IgG (1:250, Life Technology, A11008) antibody for 1 h at room temperature. Then, the coverslips or slides were washed twice with PBS and mounted with Prolong gold anti-fade reagent containing DAPI (Life Technology). In the FISH experiments, the specificity of the miR-143 signal

was confirmed via comparison with a scrambled control. Unlike the miR-143 probe, the scrambled probe showed no signal in HBMECs.

Permeability of the endothelial barrier

According to our previous study[2], primary HBMECs were plated onto Transwell inserts (0.4-µm pore size) as shown in the Supplementary information. The HBMECs were then stably transfected with the miR-143 lentivirus or anti-miR-143 lentivirus. The cells were grown for 5 days to achieve confluence, and the cultured cells were then treated with methamphetamine (100 µM). Twenty-four hours later, 200 µl of FITC-conjugated dextran-4 (1 mg/ml; Sigma) was added to the upper chamber of the Transwell plates to detect changes in monolayer permeability. After 30 min, aliquots (100 µl) were collected from the lower chamber for fluorescence measurements using excitation and emission wavelengths of 480 and 530 nm, respectively (Biotek Synergy H1 multimode microplate reader).

Chromatin immunoprecipitation (ChIP) assay

The ChIP assay was performed according to the manufacturer's instructions (Millipore, Temecula, USA) and our previous study[2,3], as shown in the Supplementary information. Fresh formaldehyde (18.5%) was added directly to the medium for cross-linking; the final concentration of formaldehyde was 1%. After a 10-min incubation at room temperature, the unreacted

formaldehyde was quenched with 10X glycine for 5 min at room temperature. Washed cells (cold 1X PBS, twice) were scraped with cold PBS containing 1X protease inhibitor cocktail and then centrifuged (800 g, 5 min, 4°C) to pellet the cells. Nuclei were harvested from the cell pellet using a lysis buffer containing 1X protease inhibitor cocktail. DNA was sheared by sonication. The sheared cross-linked chromatin was then diluted with dilution buffer, mixed with protein A magnetic beads and antibodies for p53 (Santa Cruz, sc-6243), NF-κB p65 (Cell Signaling, #3033S), STAT3 (Cell Signaling, #12640S), histone H3 (Millipore, 06-599B) and IgG (Cell Signaling, #2729), and allowed to incubate overnight at 4°C. After the cross-linked protein/DNA complexes were washed with a series of cold wash buffers, including a low-salt buffer, a high-salt buffer, an LiCl buffer, and finally, TE buffer, the complexes were reversed to free the bound DNA with elution buffer and purified using DNA purification spin columns in accordance with the manufacturer's instructions. Finally, the purified DNA was amplified via PCR to identify the promoter region containing the specific binding site. The sequences of binding sites and primer sequences are listed in Supplementary Table S1.

References

1 Zhang, Y. *et al.* Mir143-BBC3 cascade reduces microglial survival via interplay between apoptosis and autophagy: Implications for methamphetamine-mediated neurotoxicity. *Autophagy.* ***27*****,** 1-22, (2016).

2 Yao, H. *et al.* Cocaine hijacks sigma1 receptor to initiate induction of activated leukocyte cell adhesion molecule: implication for increased monocyte adhesion and migration in the CNS. *J. Neurosci.* **31,** 5942-5955, (2011).

3 Yao, H., Duan, M. & Buch, S. Cocaine-mediated induction of platelet-derived growth factor: implication for increased vascular permeability. *Blood.* **117,** 2538-2547, (2011).

Supplementary Figure S1

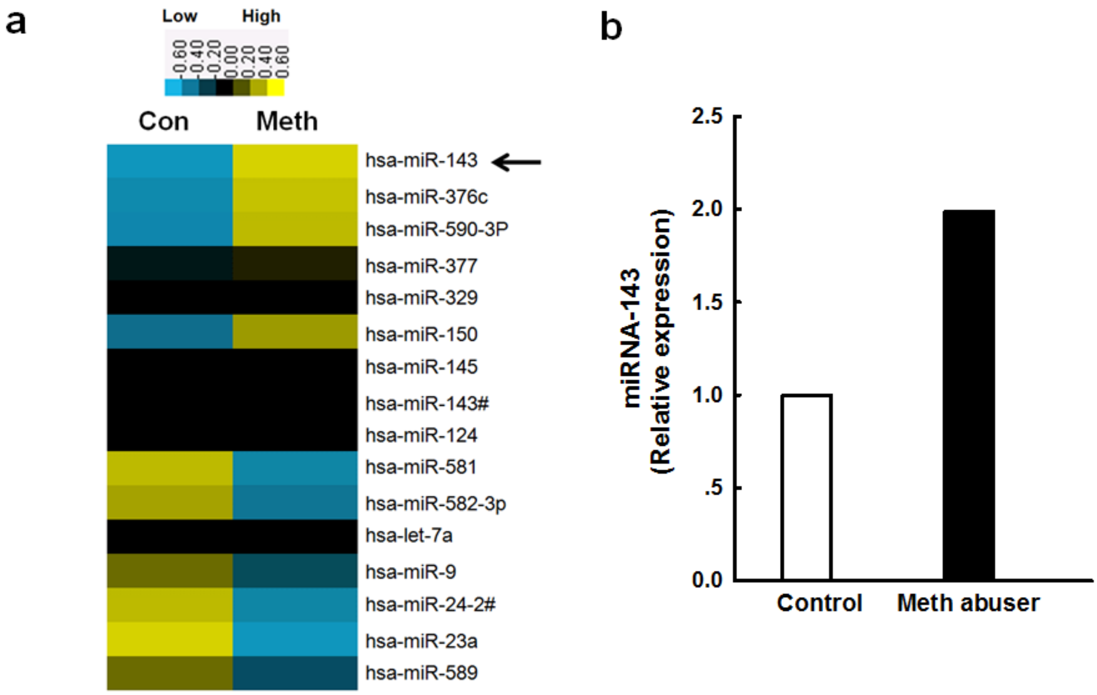

Supplementary Figure S1. Up-regulated miR-143 expression in serum from methamphetamine abusers. a) Screening of miRNAs that are differentially expressed in serum from methamphetamine abusers and the control subjects. Heat maps of the 15 most up-regulated and 15 most down-regulated miRNAs in pooled serum from methamphetamine abusers (n=17) and the control subjects (n=13). The samples represent pooled serum from 3–6 mice. All miRNAs with a reliable value (Ct<30) and a significant difference between the serum of methamphetamine abusers and their respective controls (twofold, $p<0.05$). **b)** qPCR validation of the miR-143 expression in the pooled serum from methamphetamine abusers (n=17) and from the control subjects (n=13).

Supplementary Figure S2

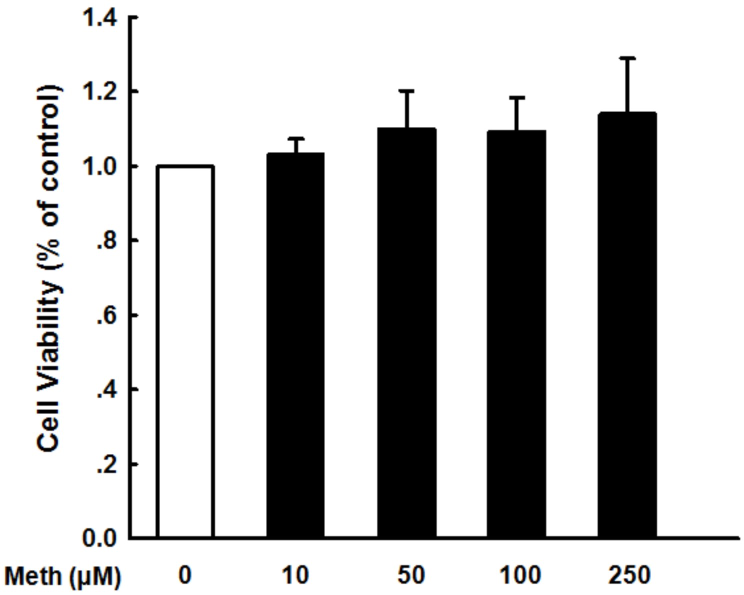

Supplementary Figure S2. Effect of methamphetamine on the viability of HBMECs. The effects of different concentrations of methamphetamine (10 μM, 50 μM, 100 μM, and 250 μM) on the viability of HBMECs were evaluated using the MTT assay. All data are reported as the mean±SD of three individual experiments.

Supplementary Figure S3

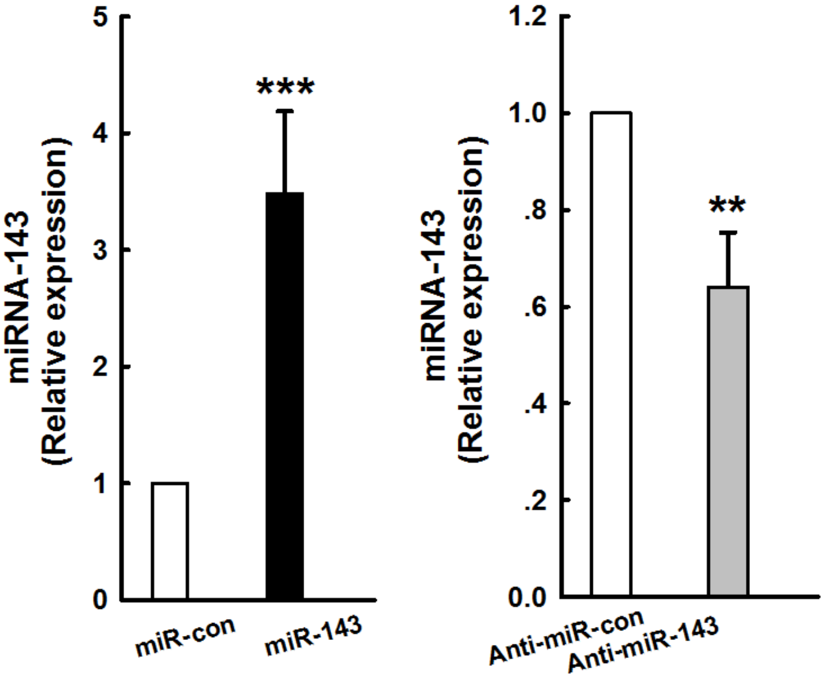

Supplementary Figure S3. Effect of miR-143 and anti-miR-143 on the level of miR-143 in HBMECs. HBMECs infected with the miR-control/miR-143 **(a)** and the anti-miR-control/anti-miR-143 **(b)** lentiviruses were assessed for miR-143 mRNA expression using real-time PCR. All data are presented as the mean±SD of three individual experiments. **p<0.01 and ***p<0.001 *vs*. the control group using Student's t-test.

Supplementary Figure S4

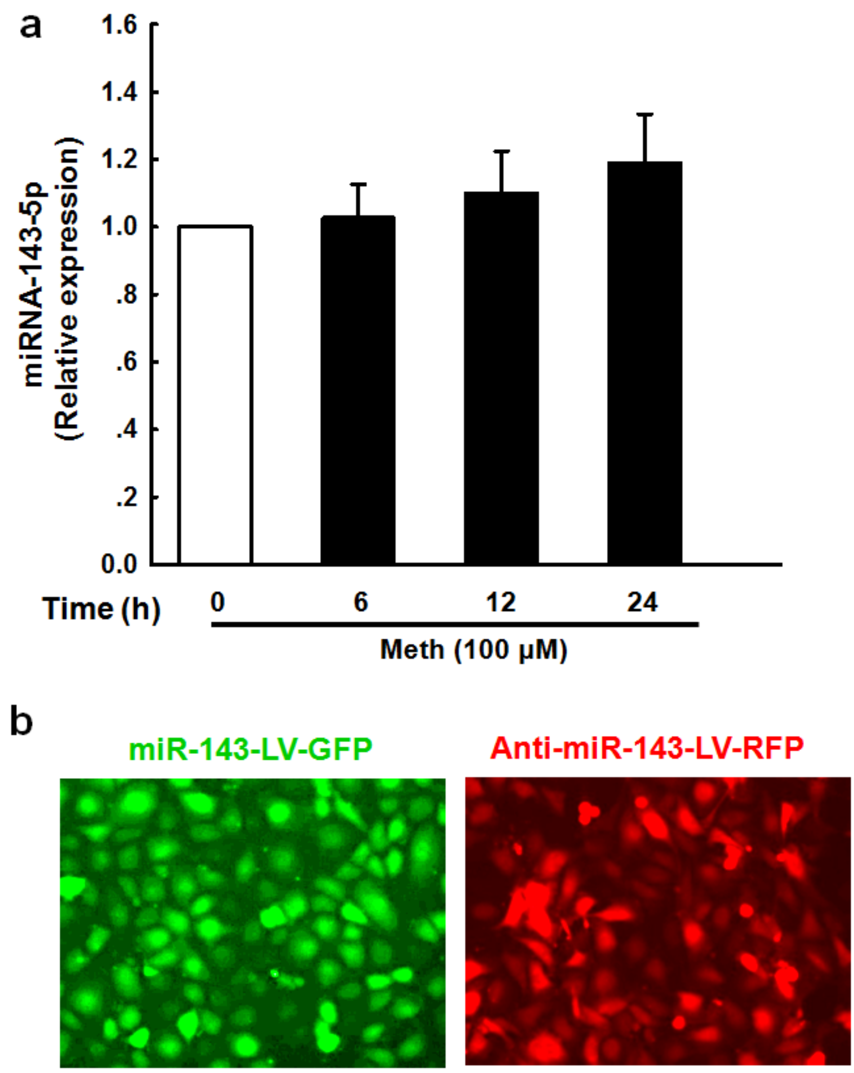

Supplementary Figure S4. Effect of methamphetamine on the expression of miR-143-5p in HBMECs. (a) HBMECs were treated with methamphetamine (100 μM) for different amounts of time, and the level of miR-143-5p expression was determined via real-time PCR. All data are presented as the mean±SD of three individual experiments. **(b)** Representative image of HBMECs transduced with miR-143-GFP (green) or anti-miR-143-RFP (red).

Supplementary Figure S5

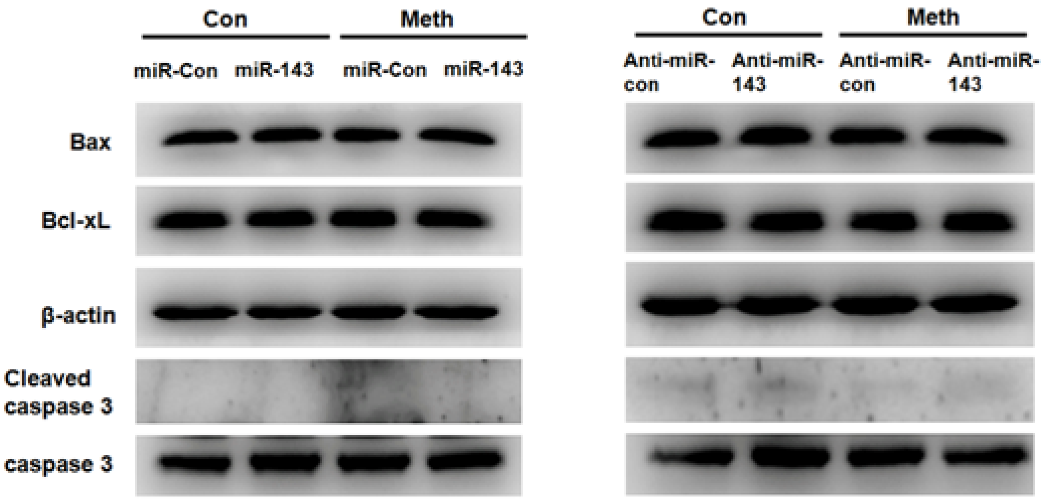

Supplementary Figure S5. Effects of miR-143 and anti-143 in HBMECs treated with or without methamphetamine. HBMECs were transduced with miR-143 or anti-miR-143 and then treated with methamphetamine for 24 h. The expression of Bax/Bcl-xL, cleaved caspase 3 and caspase 3 was evaluated using western blotting.

Supplementary Figure S6

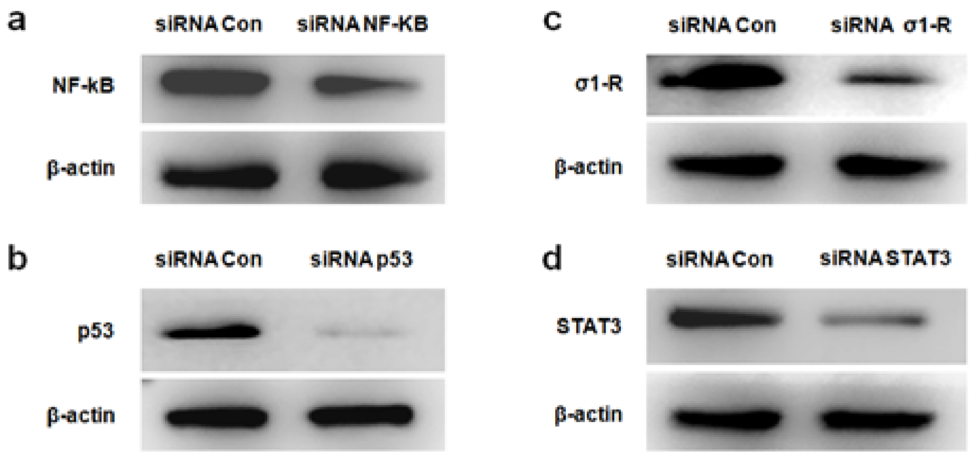

Supplementary Figure S6. siRNA-mediated knockdown of NF-κB, p53, σ-1R or STAT3 specifically and successfully decreased the expression of the corresponding protein.

Supplementary Figure S7

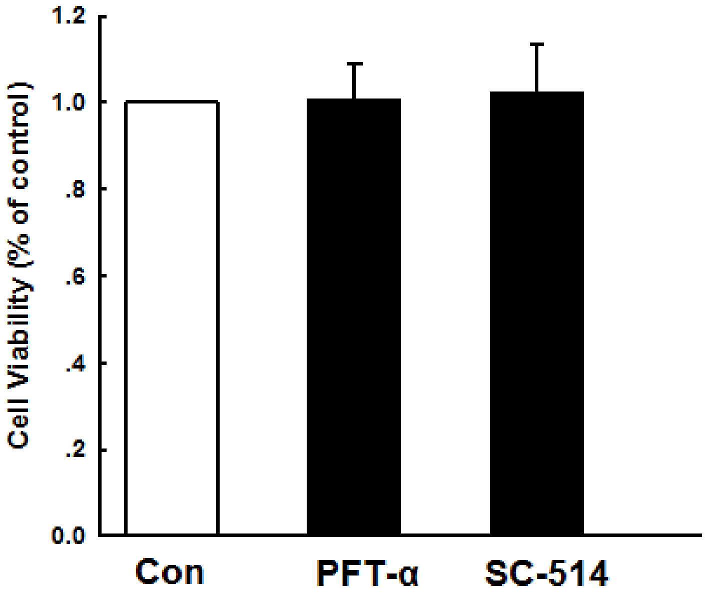

Supplementary Figure S7. Effect of the STAT3 and NF-κB inhibitors on the viability of HBMECs. The p53 inhibitor PFT-α (5 μM) and the NF-κB inhibitor SC-514 (5 μM) failed to affect the viability of HBMECs, as evaluated using the MTT assay. All data are presented as the mean±SD of three individual experiments.

Supplementary Table S1. The binding site of transcription factors for the TJPs/miR-143 and primers for identification of TJPs/miR-143 bound by p53, NF-κB or STAT3.

Gene	Transcription factor	Binding site	Primer sequences
Claudin-5	p53	GGGCAGG	5’-CGCTGTTCTTCACTTCCCCT-3’-sense<br>5’-TTAGGCAGGGACACAAACCC-3’-antisense
	NF-κB	GAGTTTCCCAA	5’- CGCTGTTCTTCACTTCCCCT-3’ –sense<br>5’-TTAGGCAGGGACACAAACCC-3’-antisense
Occludin	p53	GGGCAGG	5’-GAGAAGTGGGTGGGATTGGAT-3’-sense<br>5’-TGCCATCCAGCAGGCTAAAG-3’-antisense
	NF-κB	AGGGGAAAGTG	5’-TCATCACCCTTCAAAGCCTTCC-3’-sense<br>5’-AGATCTTGGATGCTGTGCCAT-3’-antisense
ZO-1	p53	GGGCGG	5’- TTGACCCGTTCGGTCAACAA-3’ -sense<br>5’-GAGACCTGTGACATCTCGGC-3’-antisense
miR-143	STAT3	TCCCTTTTCCCTGCAATGCTC	5’-GTGTTGCCCTGGAAAGCAAG-3’-sense<br>5’-ATGGGAGGAACAGCAGAAGC-3’-antisense
